# Altered urinary tetrahydroisoquinoline derivatives in patients with Tourette syndrome: reflection of dopaminergic hyperactivity?

**DOI:** 10.1007/s00702-020-02289-6

**Published:** 2020-12-23

**Authors:** Philipp Capetian, Veit Roessner, Caroline Korte, Susanne Walitza, Franz Riederer, Regina Taurines, Manfred Gerlach, Andreas Moser

**Affiliations:** 1 Department of Neurology, University Hospital of Wuerzburg, Würzburg, Germany; 2 Department of Child and Adolescent Psychiatry, TU Dresden, Dresden, Germany; 3 Department of Child and Adolescent Psychiatry and Psychotherapy, University of Zuerich, Zuerich, Switzerland; 4 Department of Neurology, Clinic Hietzing, Vienna, Austria; 5 Department of Child and Adolescent Psychiatry, University Hospital of Wuerzburg, Würzburg, Germany; 6 Department of Neurology, CBBM, University of Luebeck, Luebeck, Germany; 7 Department of Neurology, University Hospital Zurich, University of Zurich, Faculty of Medicine, Zurich, Switzerland

**Keywords:** Tourette syndrome, ADHD, Tics, Biomarkers, Tetrahydroisoquinoline derivates

## Abstract

Tetrahydroisoquinolines (TIQs) such as salsolinol (SAL), norsalsolinol (NSAL) and their methylated derivatives N-methyl-norsalsolinol (NMNSAL) and *N*-methyl-salsolinol (NMSAL), modulate dopaminergic neurotransmission and metabolism in the central nervous system. Dopaminergic neurotransmission is thought to play an important role in the pathophysiology of chronic tic disorders, such as Tourette syndrome (TS). Therefore, the urinary concentrations of these TIQ derivatives were measured in patients with TS and patients with comorbid attention-deficit/hyperactivity disorder (TS + ADHD) compared with controls. Seventeen patients with TS, 12 with TS and ADHD, and 19 age-matched healthy controls with no medication took part in this study. Free levels of NSAL, NMNSAL, SAL, and NMSAL in urine were measured by a two-phase chromatographic approach. Furthermore, individual TIQ concentrations in TS patients were used in receiver-operating characteristics (ROC) curve analysis to examine the diagnostic value. NSAL concentrations were elevated significantly in TS [434.67 ± 55.4 nmol/l (standard error of mean = S.E.M.), two-way ANOVA, *p* < 0.0001] and TS + ADHD patients [605.18 ± 170.21 nmol/l (S.E.M.), two-way ANOVA, *p* < 0.0001] compared with controls [107.02 ± 33.18 nmol/l (S.E.M.), two-way ANOVA, *p* < 0.0001] and NSAL levels in TS + ADHD patients were elevated significantly in comparison with TS patients (two-way ANOVA, *p* = 0.017). NSAL demonstrated an AUC of 0.93 ± 0.046 (S.E.M) the highest diagnostic value of all metabolites for the diagnosis of TS. Our results suggest a dopaminergic hyperactivity underlying the pathophysiology of TS and ADHD. In addition, NSAL concentrations in urine may be a potential diagnostic biomarker of TS.

## Introduction

Tics are short, non-rhythmic and non-purposeful single or short series of movements or vocalizations. Suppression can be achieved for a short time but the build-up of so-called “premonitory urges” finally enforce the execution of a tic to release the pressure (Leckman et al. 1993). Transient tics occur during childhood and adolescence in up to 15% of all healthy children (Robertson 2008). Persistence of tics for more than one year defines a chronic tic disorder, and the combination of chronic vocal and motoric tics is termed Tourette syndrome (TS) (American Psychiatric Association 2013). TS is typically associated with comorbid psychiatric conditions such as attention-deficit/hyperactivity disorder (ADHD), obsessive compulsive disorder, anxiety and depression.

The etiology and pathophysiology underlying TS are still largely unknown. However, there is some evidence for a dopaminergic hyperinnervation in TS causing increased tonic and phasic dopaminergic neurotransmission in the basal ganglia (Maia and Conceição 2018). The efficacy of dopamine antagonists in the treatment of TS (Pringsheim et al. 2019) and exacerbation of tics by dopaminergic drugs like dextroamphetamine (Erenberg et al. 1985; Cohen et al. 2015) underscore the relevance of this neurotransmitter system in this condition.

**Fig. 1:**
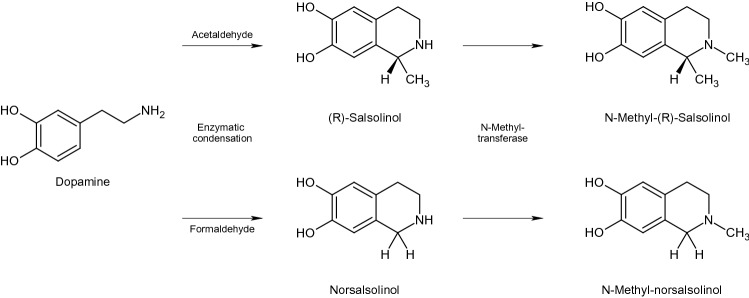
Biosynthesis of tetrahydroisoquinoline derivatives from dopamine

The dopamine-derived tetrahydroisoquinolines (TIQs) (R)-salsolinol (SAL) and norsalsolinol (NSAL) are synthesized in the human brain from dopamine either by a non-enzymatic *Pictet*-*Spengler* reaction or enzymatically via salsolinol synthase (Fig. 1) (Moser 1998b). SAL and NSAL are metabolized by N-methyl-transferase into their N-methyl-derivatives N-methyl-salsolinol (NMSAL) and -norsalsolinol (NMNSAL) (Fig. 1) (Moser 1998b). There is evidence that these derivatives are involved in central dopamine metabolism (Briggs et al. 2013) and in the modulation of dopaminergic neurotransmission (Xie et al. 2012). In a previous study, we found higher concentrations of all these dopamine-derived metabolites in urine of ADHD patients, a condition also discussed to be associated with a disturbed dopamine neurotransmission (Roessner et al. 2007).

The aim of this study is to measure for the first time the concentrations of TIQs in the urine of patients with TS and patients with comorbid ADHD compared with controls. In addition, we want to explore the value of TIQs regarding the diagnosis of TS.

## Methods

### Subjects

The local ethics committee of the participating universities approved the study protocol. Seventeen children, adolescents and young adults fulfilling the diagnostic criteria of chronic tic disorders (F95.1) including TS (F95.2) as referred to by the international classification of diseases in its 10th edition (ICD-10) (World Health Organization and Staff 1992) and 12 patients with this condition and a comorbid ADHD were included in this study. All patients were screened and diagnosed by a board-certified child and adolescent psychiatrist through a semi-structured interview supported by diagnostic checklists. The characteristics of the study cohort are shown in Table 1.

**Table 1:** Demographic characteristics and psychopharmacological prescription of patients and controls taking part in the study

	Controls	TS	TS + ADHD
*Demographics*			
Subjects included	19	17	12
*Subjects per site*			
Goettingen	8	13	6
Wuerzburg	11	4	6
M/F	10/9	13/4	11/1
Mean age ± S.E.M.	11.21 ± 1.1 y	13.04 ± 0.83 y	13 ± 0.95 y
Age range	5–20 y	6–22 y	7–19 y
*Medication*			
Biperiden	0/19	0/17	2/12
Risperidone	0/19	17/17	2/12
Stimulants	0/19	0/17	10/12
Tiapride	0/19	4/17	2/12

Patients were recruited in two sites (Departments of Child and Adolescent Psychiatry of the University Hospitals of Wuerzburg and Goettingen). The patients and their parents or legal guardians gave informed consent.

The control group was recruited from 19 healthy children and adolescents without any medication (either relatives of hospital employees or from a school class in Goettingen). A child and adolescent psychiatrist evaluated all participants in the control group to rule out the presence of psychiatric disorders.

### Urine collection and analysis

Due to the presence of high levels of TIQ derivatives in certain types of food and beverages, participants were instructed to omit fresh and dried banana, cheese, chocolate soy sauce, beer, port and white wine for 48 h before and during the sample collection. Psychostimulants were discontinued 48 h before the urine collection and reinitiated after the collection. Intake of the other medication was continued during the study. Urine was collected starting at 7 pm over 12 h in the presence of 50 mg semicarbazide and 50 mg Na_2_EDTA. After collection, samples were aliquoted and stored at − 40 °C until further analysis.

Samples were analyzed three times for all conditions by blinded staff. Analysis consisted of a two-stage process: (1) processing by affinity chromatography, (2) quantification of TIQ derivatives by high-performance liquid chromatography with electrochemical detection (HPLC/ECD) as previously described (Moser et al. 1996a). A rigorous quality control routine was employed by adding negative controls (water and urine of healthy persons with no measurable TIQ excretion) and positive controls (water and urine of healthy persons with all 4 TIQ added at 1 nmol/ml).

As described earlier (Moser 1998b), dihydroxylated TIQ derivatives are in part bound to sulfo- or gluco-residues. Bound metabolites can only be measured after deconjugation by incubation with arylsulfatases and β-glucuronidases. For this study, only free unconjugated metabolites were measured because conjugated metabolites are not able to modulate dopaminergic neurotransmission (Moser 1998b). SAL and NSAL are present in two enantiomeric forms ((S)-SAL and N-(S)-SAL as well as (R)-SAL and N-(R)-SAL (Moser 1998a)). The analytic process did not differentiate between the two forms.

### Statistics

Statistical analysis was performed with the GraphPad Prism 8.02 software (GraphPad Software, San Diego, USA).

Data were expressed as mean values ± standard error of mean (S.E.M.). In order to rule out statistically significant differences in the mean age of the groups, an ordinary one-way ANOVA with Tukey’s multiple comparisons test was performed. Differences in free urinary TIQ levels were analyzed by two-sided analyses of variance (two-way ANOVA) followed by the Sidak’s multiple comparison test. In order to determine the diagnostic value of individual TIQs for TS, the receiver-operating characteristics (ROC) curves including areas under the curve (AUC) were calculated for free urinary levels between the control and TS group.

## Results

**Fig. 2:**
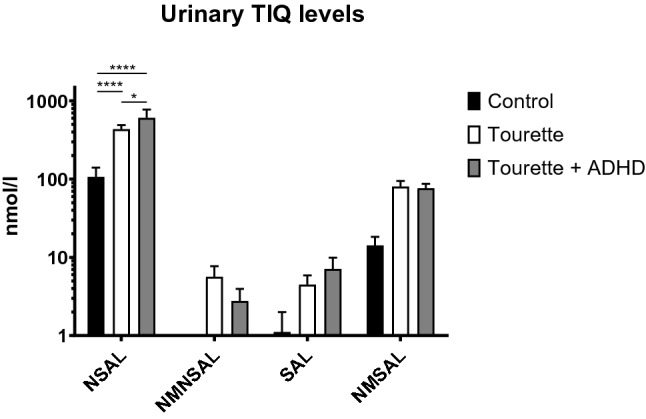
Urinary levels of free tetrahydroisoquinoline derivatives in patients with chronic tic disorders including Tourette syndrome (TS), TS with comorbid attention-deficit/hyperactivity disorder (TS + ADHD) and age-matched healthy controls. (*n* = 17 for TS, n = 12 for TS + ADHD and *n* = 19 for controls, error bars represent standard error of the mean, two-way ANOVA with Sidak’s multiple comparison test, logarithmic scale). Please note: NMNSAL could not be detected in any control subject, therefore no corresponding bar is present in the graph

All mean values of free urinary TIQ levels were higher in patients with TS and comorbid ADHD than in controls (Fig. 2). However, statistically significant differences were observed only for NSAL between TS and controls (*p* < 0.0001), TS + ADHD and controls (*p* < 0.0001) as well as TS and TS + ADHD (*p* = 0.017).

To determine the diagnostic qualities of TIQ values in respect of a diagnosis of TS, ROC curves including AUC were used. NSAL (0.93) and NMSAL (0.91) demonstrated higher AUC values than SAL (0.71) and NMNSAL (0.67). P values reached significance for NSAL (*p* < 0.0001), SAL (*p* = 0.04) and NMSAL (*p* < 0.0001).

## Discussion

We found higher urinary concentrations of NSAL in TS patients compared with controls. Interestingly, the highest levels were measured in patients with TS and comorbid ADHD. A similar trend was observed for SAL (Fig. 1). These findings support the notion of a “hyperdopaminergic” state in TS (Maia and Conceição 2018).

ADHD has already been described before as a condition associated with elevated TIQ levels (Roessner et al. 2007). This assumption is strengthened by the fact that the original publication on elevated TIQ levels in ADHD patients (which recruited its patients in the same centers) exhibited the most pronounced elevation for NSAL as well. Furthermore, the mean NSAL level of ADHD patients in the mentioned study was 553.3 nmol/l and thus between the mean levels of TS patients (434.67 nmol/l) and TS + ADHD patients (605.18 nmol/l) in the present study. It seems consequential that patients suffering from two conditions associated with a potential hyperdopaminergic state to have even higher TIQ levels than patients with only one of those.

TIQs are metabolized either directly from dopamine (NSAL and SAL) or from each other (NMNSAL from NSAL and NMSAL from SAL). TIQ levels were expressed as nmol/ml, a unit which showed the best correlation with derivates of the dopamine metabolism (homovanilic acid (HVA) and MDOPA) in the cerebrospinal fluid (CSF) of humans (Moser et al. 1996b).

Individual metabolites exhibit big differences between measured levels in the range of up to several 100 × and the differences are not necessarily consistent: NSAL levels in our study are higher than NMNSAL levels that are metabolized from them. However, NMSAL derived from SAL exhibits higher levels. We hypothesize that different affinities of NSAL and SAL to the metabolizing enzymes (none of which have been characterized in detail for TIQs to our knowledge) could be the reason. On the other hand, the differences between controls and TS or TS + ADHD for all individual metabolites are constantly in a magnitude between fourfold and sevenfold. This fact increases in our opinion the likelihood of altered metabolite levels between controls, TS and TS + ADHD as consequence of differences in catecholamine levels. As TIQ derivatives can cross the blood–brain barrier, urinary levels can be expected to correlate with the levels in the brain and it has been demonstrated that they correlate with dopamine metabolites in the CSF (Moser et al. 1996b).

**Fig. 3:**
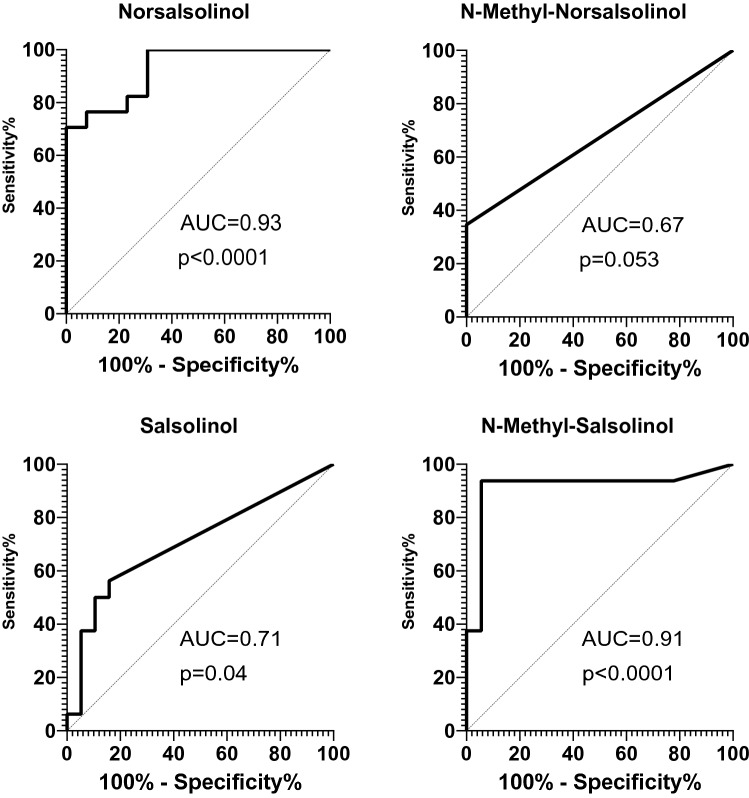
Receiver operator characteristics (ROC) curve analyses of free urinary TIQ levels. N-salsolinol demonstrates the highest area under the curve (AUC) and thus the best discriminative properties of all studied TIQ metabolites

Our study demonstrated that TIQ metabolites might even carry a diagnostic value for TS. The metabolite with the biggest difference between TS and controls (NSAL) demonstrated a particularly high AUC. The diagnosis of tics and TS relies solely on clinical observation. The only study so far on the validity of TS diagnoses compared the prima vista diagnosis made by two movement disorder experts with the clinical diagnosis made by a much more comprehensive process in clinical routine in a tertiary center. It demonstrated a correct diagnosis in 71% and 96% of cases based on a 20 s video of the individual tics with an inter-rater reliability of 0.71 (Paszek et al. 2010). Thus, clinical diagnosis is not flawless. However, if future studies might correlate TIQ levels to the severity and/or frequency of the occurrence of tics, they could become a valuable follow-up parameter for therapies and potentially select patients who might benefit from an anti-dopaminergic therapy (Fig. 3).

A major potential confounder represents the ongoing medication of the patients. Due to their short half-life, psychostimulants were discontinued 48 h before the urine collection. Intake of the antipsychotic drugs (risperidone, tiapride) used to treat TS was not discontinued because of the longer time it would have taken for washout and reinitiating. Although it was shown in a positron emission tomography study in healthy subjects that oral administration of a single dose does not change the dopamine synthesis capacity (Ito et al. 2009), it cannot be ruled out that chronic antipsychotic treatment affects central dopamine levels. In addition to the blockade of postsynaptic dopamine D2 receptors, antipsychotics modulate presynaptic function. For example, increased dopamine release and increased dopamine metabolism have been reported following antipsychotic treatment in animal studies (Park et al. 2019).

Psychiatric comorbidity represents another potential confounder. ADHD is the most common comorbid condition in TS (El Malhany et al. 2015) and was therefore included as an individual patient group in this study. We cannot fully rule out that the other comorbidities present in roughly 16% of TS and 25% of TS + ADHD patients in this study are associated with altered TIQ levels themselves and might alter TIQ levels.

As already mentioned, TIQs measured in the patient’s urine might as well come from alimentary sources. We therefore instructed patients to follow dietary restrictions on certain types of food and beverages and thus minimizing this confounder in our study. However, there was no possibility to objectify the adherence of individuals partaking in this study.

TIQ levels were measured in urine from a 12 h collection. This serves as a measure to compensate for potential diurnal changes in metabolite levels and their excretion. The completeness of the collection is fully dependent on the compliance of individuals in the study.

## Conclusions

We found elevated levels of NSAL in the urine of TS patients with and without comorbid ADHD in comparison with age-matched healthy children and adolescents. These results suggest higher dopamine synthesis in TS but have to be confirmed in studies with drug-naïve TS patients. NSAL as the metabolite with the biggest increase compared with controls might also carry a diagnostic value for identifying patients with TS.

## Data Availability

Source data are available upon request.
